# A Systematic Underpinning and Framing of the Servicescape: Reflections on Future Challenges in Healthcare Services

**DOI:** 10.3390/ijerph15030509

**Published:** 2018-03-13

**Authors:** Jieun Han, Hyo-Jin Kang, Gyu Hyun Kwon

**Affiliations:** Graduate School of Technology and Innovation Management, Hanyang University, Seoul 04763, Korea; juliahanje@hanyang.ac.kr (J.H.); hyojin.sara.kang@gmail.com (H.-J.K.)

**Keywords:** servicescape, healthscape, physical environment, built environment, healthcare service, service evaluation, systematic literature review

## Abstract

Ever since Bitner defined the term “servicescape” as the physical environment in which the service is assembled, several scholars have attempted to better understand the impact of the built environment in the context of different service settings. While servicescape is a topic of increasing academic interest among scholars and practitioners, most studies in the area are dedicated to understanding the built environment of hedonic service. More studies are needed to examine utilitarian servicescape and in this paper, we have focused on the healthcare environment. This study aims to identify the gap in servicescape and healthscape studies by providing a theoretical structure of the current servicescape literature and comprehend the academic differences between hedonic servicescape and utilitarian healthscape studies. After reviewing 44 selected papers based on rigorous criteria, we: (1) framed the servicescape factors; (2) analyzed the servicescape literature from the perspectives of terminologies, research fields, methodologies, and frameworks; and (3) identified the current paths of healthscape research. Through this work, we highlight the significance of adopting different dimensions and factors to evaluate the distinguished service environment by the servicescape type and propose several research agendas for future studies on healthscapes. The research findings can contribute to a deep understanding of healthscapes and can introduce a new viewpoint for interpreting the servicescape in diversified service settings.

## 1. Introduction

The services industry contains intangibility, inseparability, and heterogeneity [1], making it extremely difficult to measure service quality. People depend on more tangible clues. In other words, the space where the service is produced cannot be hidden and may have a strong effect on customers’ perceptions of the service experience [2,3,4,5,6,7,8,9,10,11]. Following this point, Bitner defined the term “servicescape” as the physical environment in which the service is assembled [2]. The scholar attempted to explain three servicescape dimensions (ambient conditions, spatial layout and functionality, and signs/symbols and artifacts) and their interactions with customers and service providers. Other scholars adapted this theoretical framework to different service settings, for instance, leisure services [3,4], restaurants [12,13,14], hotels [5], museums [7], and airports [15].

Healthcare service providers also have realized the significance of the physical environment to enhance their service quality. Hutton et al. suggested the term “healthscape” as the servicescape of healthcare services and explained how the healthscape affects a patient’s perception, attitude, satisfaction, and behavioral intention [16,17,18,19,20].

As the types of servicescapes have diversified from retail services to healthcare services, aggregating the results of relevant research in the field and providing an overview of the current status of servicescape studies would contribute significantly to the literature. Literature reviews related to servicescapes have been conducted by several researchers. Turley and Milliman focused on constructing a comprehensive table of the empirical studies regarding the effects of physical environments in commercial service contexts [21]. They identified atmospheric variables and suggested a framework of the servicescape for the retail service environment. Lin attempted to review the literature relating to the impact of the servicescape on customer behavior, based on the concept of Gestalt psychology [6]. The author proposed a framework for a customer’s evaluation process for a servicescape and explained each step by analyzing the existing literature. Another study by Aubert-Gamet et al. noted that the impact of a servicescape was still very uncertain and researchers should consider the emergence of a new social condition with paramount consequences on consumer behavior [22]. Moreover, Mari et al. insisted that we still lack an up-to-date systematization of both theoretical and empirical findings on how servicescapes affect customer behavior [23].

Although scholars have attempted to explain the transition of servicescape studies in their studies, several have insisted that research on servicescapes has some limitations, such as: (1) servicescapes of diverse service settings cannot be generalized since several initial studies focused only on the hedonic service and (2) some of the dimensions (factors) of servicescapes do not reflect emerging social changes because of intangible spaces, such as cyberspace [13,16,24,25,26,27]. From this perspective, classifying the results of servicescape studies between hedonic and utilitarian domains would be significant to explain the characteristic properties of the service environment.

The principal objective of this paper is to: (1) provide an overview of the current status of servicescape research with diverse viewpoints; (2) identify the differences between hedonic servicescape and utilitarian healthscape studies by classification; and (3) illustrate the academic challenges of healthscape research. The results of this study could support the understanding of overall theoretical boundaries on servicescape and create opportunities for further healthscape studies.

The remainder of this paper is organized as follows: Section 2 addresses the theoretical background of the servicescape and healthscape. Section 3 focuses on the research methodology, Section 4 provides research findings, and Section 5 presents the discussion and implications. Finally, Section 6 presents the conclusions of the study.

## 2. Previous Research

### 2.1. Servicescape

Studies on the physical environment of service organizations are based on the research of Kotler and Baker [28,29]. Kotler defined “atmospherics” as the physical and controllable environmental components affecting the buyer’s purchasing probability [28]. He argued that the atmosphere could affect purchase behavior since it may directly arouse visceral reaction that favorably contributed to purchase probability. Baker also highlighted the role of the environment in marketing services and how the physical environment influences customer perceptions of service [29]. Drawing from these two perspectives, Bitner suggested the term “servicescape,” referring to “the physical surroundings that impact on the behaviors of customers and employees in service organizations” [2]. She identified that both customers and employees perceive their service environment through a variety of objective environmental factors and both groups may respond cognitively, emotionally, and physiologically to the environment. Those internal responses to the environment influence the behavior of individual customers and employees in the servicescape and affect social interactions between and among customers and employees. She identified three environmental dimensions: (a) ambient conditions; (b) spatial layout and functionality; and (c) signs, symbols, and artifacts. Ambient conditions refer to the characteristics of the environment that can stimulate the five senses of customers (temperature, lighting, noise, music, and scent). Spatial layout refers to the size, shape, and arrangement of machinery, equipment, and furnishings, whereas functionality refers to the ability of these items to fulfill customer needs. Lastly, signs, symbols, and artifacts indicate tools that can provide customers with information about service facilities.

After Bitner’s study, several scholars adopted the servicescape framework and tried to explain their studies by developing their own environmental factors for different types of service settings (see Appendix A). For instance, Wakefield et al. implemented a survey of leisure service settings in the USA and identified five environmental dimensions: layout accessibility, facility aesthetics, seating comfort, electronic equipment, and facility cleanliness [30]. In another study on restaurants, Raajpoot suggested the term “tangserv” and found that the physical environment should be considered in terms of layout/design, product/service, and ambient/social [12]. Similarly, in the context of museums, Kottasz categorized environmental factors as exterior factors, interior factors, layout and design, decorations, and human factors [7]. Lee conducted a study on festival services and proposed that the attributes of a physical environment should include program contents, staff, facility, food, souvenirs, convenience, and information [27].

### 2.2. Servicescapes in the Healthcare Industry: Healthscapes

The healthcare service industry could be distinguished from other retail service sectors, as it is difficult for patients to estimate and judge the service they are provided. It also needs professional knowledge of medical information and experience [16,20,31]. As a result, tangible clues, such as the physical environment, etc. play a major role in evaluating the entire healthcare service.

Originating from the domain of the servicescape, Hutton et al. pointed out that despite the paramount and permanent importance of the physical facility [16], little research has been accomplished involving the marketing role that the healthcare facility and physical environments play [16,20,31]. They proposed the term “healthscape” and identified components of atmospherics and servicescapes concerning healthcare to assess their strengths and predictive abilities in the relationships between patient satisfaction and quality assessment, intention to return, and willingness to recommend a healthcare provider to others and to propose much needed research in the area. Healthscape can be defined as the emotional, affective, cognitive, and physiological influence on the patient-customer and staff-provider behaviors and outcomes caused by elements of the physical healthcare environment, including the facility and tangible elements of the service encounter.

The concept of healthscape motivated researchers to face the academic challenges in investigating the effect of the healthscape on patients. For instance, Lee explored the conceptual framework of healthcare facilities from a service design perspective based on Bitner’s study [18]. She discovered that healthscape factors could be placed into one of two categories: ambient conditions and serviceability. These factors are related to the satisfaction of patients, perceived quality of care, and approach behavior. Another study conducted by Rosenbaum et al. identified that perception of the healthscape could create restorative effects on patients in cancer resource centers [19]. They proposed a theoretical understanding of how physical factors have an impact on patients to relieve their stress. Sahoo et al. identified six healthscape factors that led to patients’ satisfaction in Indian private healthcare facilities through an empirical study [20]: (1) service personnel conduct and cleanliness; (2) service delivery; (3) ambience and facilities; (4) location and look; (5) appealing decoration; and (6) upgraded safety service. This result implied that dimensions of the healthscape should be considered separately from those of the general commercial service sector.

## 3. Method

This study has conducted a systematic literature review based on the guidelines of the PRISMA process [32,33]. No meta-analyses were conducted, due to the diversity of service typologies. The steps in the systematic literature review method are provided below.

### 3.1. Research Perspectives

We conducted a systematic literature review of servicescape studies to identify research trends since Bitner’s findings. The research perspectives addressed in this study are:

What are the key terminologies used to explain the servicescape and what academic field journals mainly deal with servicescape studies?

Which research method is used to evaluate the servicescape?

What variables are used to explain the framework of the servicescape?

What are the evaluation factors of the servicescape and are there any differences according to the service setting?

Based on these criteria, we examined the current status of healthscape studies with contextual points of view using descriptive statistics.

#### 3.1.1. Databases Searched

The electronic databases searched in this review included original qualitative and quantitative papers published in Science Direct, Scopus, and Web of Science. The journals were selected, as they were known to include either empirical studies or literature reviews in the area of physical science and social science.

#### 3.1.2. Keywords Searched

The search terms that we used were not only “servicescape” but also some terms that are indicative of the physical environment of service settings. They were “service setting,” “service environment,” “atmosphere,” “physical surroundings,” “physical evidence,” “physical environment,” “spatial design,” and “built environment.” All research papers that included the above words in keywords, titles, and abstracts were considered.

#### 3.1.3. Selection of Research Papers for Inclusion in the Review 

A number of further selection criteria were specified to include appropriate studies in the review. To be included in the review, papers were required to meet all of the following inclusion criteria: (a) peer-reviewed articles/journals; (b) accessed via electronic search; and (c) written in English. According to these three conditions, 940 research papers met the inclusion criteria and were identified as relevant to the current review.

**Figure 1:**
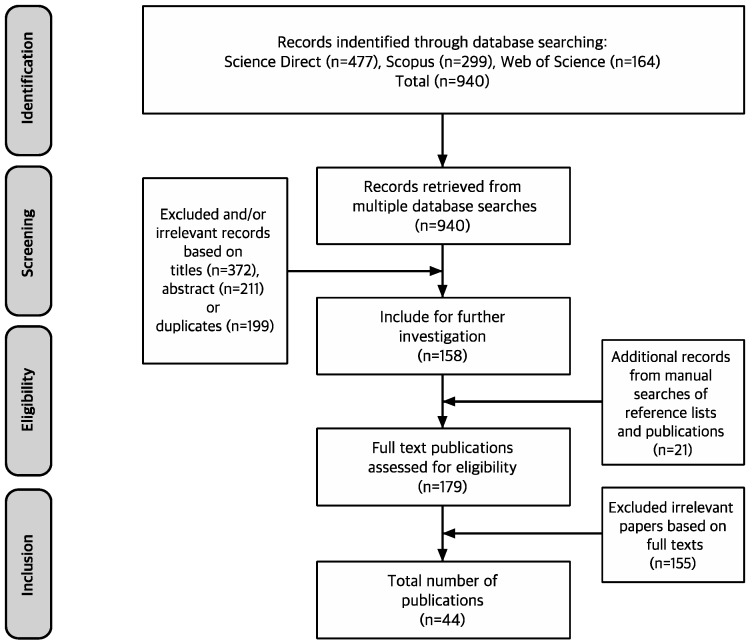
Flow diagram of the screening method to consider journals for inclusion in this literature review.

Three researchers screened each publication and excluded irrelevant journals based on titles (*n* = 372), abstracts (*n* = 211), and duplicates (*n* = 199). In this process, journals that mainly studied invisible spaces (i.e., cyberscapes, virtualscapes, online environments, musicscapes, and soundscapes) and social interactions between customers and servicescapes (i.e., socialscapes) were excluded. Whether social interaction would belong to the boundary of servicescape research is a controversial issue among scholars. In this paper, we included servicescape studies which mainly discussed physical environments or tangible elements. Furthermore, we performed a secondary investigation to include additional journals obtained from manual searches of reference lists and publications (*n* = 21) and excluded irrelevant studies based on the full text with the following exclusion criteria (*n* = 155): (a): drawn insights on servicescape is trivial or general since these papers do not discuss the physical service environment as a major construct to satisfy customers; (b) those studies that interpreted and analyzed the concepts of servicescape regardless of Bitner’s findings [2] and do not take an approach to tangible settings. Finally in total, we could extract 44 studies as shown in Figure 1.

### 3.2. Data Analysis

#### 3.2.1. Classification of the Servicescape Typology

Bitner suggested a typology of organizations based on variations in form and usage of the servicescape [2]. It is classified by stakeholder (self-service, interpersonal service, and remote service) and physical complexity (elaborate and lean). Although many studies after Bitner have been conducted, most of them are positioned in one category: elaborate—interpersonal service. As we tried to classify the literature concerning servicescapes and compare articles in the area, further classification criteria were needed. Lee et al. defined four types of servicescapes based on touch points for different service settings as follows [34]:

Type 01: intangible and hedonic (e.g., hotel, resort, and airline)
Type 02: tangible and hedonic (e.g., cinema, restaurant, and department store)
Type 03: intangible and utilitarian (e.g., hospital)
Type 04: tangible and utilitarian (e.g., discount department and gas station)

In this study, we categorized the servicescape typology used in all existing literature by the subject of the journal or data samples. To categorize studies that dealt with a general service setting, we created a group called “overall service setting.”

#### 3.2.2. Classification of Article Types

In this study, we categorized article types as follows:

TC: theoretical and conceptual papers
MP: modeling papers
LR: literature reviews
DF: dimension/factor analysis

#### 3.2.3. Classification of Servicescape Factors

Developing dimensions by service setting is one of the fundamental requirements to study the servicescape. Turley et al. stated five atmospheric variables as follows [21]: (a) external variables; (b) general interior variables; (c) layout and design variables; (d) point-of-purchase and decoration variables; and (e) human variables. We developed other criteria since the dimensions of Turley et al. are focused only on the retail environment [21]. Although several scholars have attempted to suggest their own category of dimensions, some of them are duplicated or are not considered, as they have not been reviewed against the respective attributes or items.

**Figure 2:**
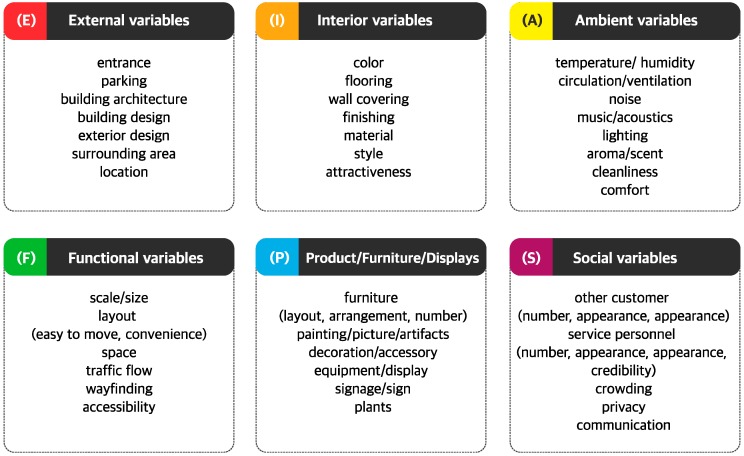
Classification of the six servicescape dimensions and factors.

We conducted affinity analysis of the groupings of servicescape factors using the bottom-up method. After taking all attributes from the 44 studies, relative items were arranged in similar categories. Three researchers conducted this work simultaneously and compared their results with each other. To assess the inter-rater reliability with respect to the quality classifying factors, a sub-sample was coded independently by researchers. The inter-rater reliability for the total results was 0.91, showing a good agreement among the three coders on the quality of the papers. Therefore, we defined six groups of service factors that could adapt to all kinds of service settings: external variables, interior variables, ambient variables, functional variables, product/furniture/displays, and social variables. The classification of the servicescape factors is shown in Figure 2 below.

## 4. Research Findings

### 4.1. Academic Evolution of the Servicescape Literature

#### 4.1.1. Terminology Used

Since Bitner defined servicescapes as “the physical surroundings (built environment) that impact on the behaviors of customers and employees in service organizations,” [2] various studies have been conducted in different service settings, from hedonic spaces (i.e., casinos and restaurants) to utilitarian spaces (i.e., hospitals and gas stations). According to the domain of service, researchers identified specific terms by adding the particular suffix (-scape). Table 1 shows the diverse academic terms that illustrate the concept of the servicescape.

By referring to Table 1, we could determine the servicescape terminology used recently, as existing papers in the literature are numbered in chronological order. “Servicescape” and “physical environment” were the main and most recently used terminologies that explain the built environment of service settings. The term “healthscape,” credited to Hutton et al., is considered official academic terminology and will be used to identify the servicescape of healthcare facilities henceforth [16].

#### 4.1.2. Research Fields of Servicescape Literature

Research fields were classified according to the names of the journals. As a result, most of the studies that are related to servicescape were discussed in marketing/business/service (50%, *n* = 22) and tourism/hotel journals (27%, *n* = 12) (See Table 2). Even though the main concept of servicescape dealt with the physical environment, results indicated that design/architecture journals (9%, *n* = 4) rarely examined the service setting. Healthscape journals, in a similar manner, mostly published healthcare and marketing/business/service sector studies (6%, *n* = 3).

#### 4.1.3. Research Methodology

Analyzing the research focus of journals could aid comprehension of how researchers have conducted their servicescape studies. As shown in Table 3, half of all the studies deal with the MP/DF type (50%, *n* = 22) compared to the TC type (11%, *n* = 5) and the TC/LR type (4%, *n* = 2). In other words, most of the researchers have examined which factors were important and how those factors influenced the servicescape in various service settings, rather than establishing or developing the concept of the servicescape. Relatively speaking, healthscape studies have been implemented at every level, from the TC to the MP/DF.

### 4.2. Framework of the Servicescape

#### 4.2.1. Independent Variables 

Examining independent variables affected by servicescape factors could help understand the entire process of functioning of the servicescape. If one of the main goals of a marketing strategy was defined as satisfying customers [35,36,37], diverse positive emotions and reaction factors may be investigated as independent variables of the servicescape. Most of the journals analyzed influencing relationships on behavioral responses (i.e., behavioral intentions and approach/avoidance) or patronage intentions (i.e., repatronage intentions, recommendation, and positive WOM), and the servicescape. In addition, resource expenditure (i.e., money/time spent and desire to stay) and loyalty intentions were independent variables of the servicescape. Table 4 shows the framework of servicescapes.

#### 4.2.2. Special Mediating/Moderating Variables

According to the type of service setting and research area, diverse mediating/moderating variables were created. Kim et al. found that restaurant type had a moderating role among perceived service quality, pleasure-feeling, and revisit intention [38]. Moreover, Ali et al. suggested national identity as a moderating factor to evaluate the physical environment for users’ delight and satisfaction [11]. External factors, such as price and social factors, such as customer familiarity, were special mediating/moderating variables when evaluating service settings [39,40].

#### 4.2.3. Stakeholder Perspectives

Researchers who have conducted studies on servicescapes predominantly conducted their research from the perspective of the customer (86.4%), rather than that of the service provider/staff (6.8%). If we recall, the original meaning of the servicescape has mainly been presented from the point of view of the end-user rather than that of the service provider/staff, and these results should naturally be evaluated. Nevertheless, recent studies pointed out the significance of the service environment for the service staff since they spend a lot of time with customers and there were fewer opportunities to renovate the environment for the service staff alone [41].

### 4.3. Factor Classification by Servicescape Typology

Table 5 below shows the number of frequencies in each of the papers that we identified using our criteria, given in Figure 1. The frequency was counted as the total number of attributes divided by the number of articles and converted in to a percentage. This numerical value could provide a clue to find out which dimensions of servicescape are addressed predominantly. Each of the papers was categorized by servicescape typology and the mean value was calculated using servicescape factors (total frequency/the number of papers).

#### 4.3.1. Overall Service Setting

Among the 44 papers, there were five studies that dealt with overall service organizations. Clearly, most factors were found in ambient variables (300%, *n* = 15), followed by interior variables (180%, *n* = 9), and then followed by product/furniture/displays (140%, *n* = 7). If we excluded peaks (ambient variables) and valleys (exterior variables), every factor was between 100% and 200%. This could be interpreted as equally important, without any noticeable difference.

#### 4.3.2. Intangible and Hedonic Servicescape (Type 01)

Intangible and hedonic servicescapes were discussed in 15 papers. These were implemented at hotels, casinos, airlines, and sports stadiums. The most frequently represented in the data were product/furniture/displays (333%, *n* = 50), followed by functional variables (260%, *n* = 39), and ambient variables (207%, *n* = 31). This meant that tangible clues (i.e., display panels, score boards, digital devices, and seating areas) of the service environment could be a key factor when people evaluate the whole service.

#### 4.3.3. Tangible and Hedonic Servicescape (Type 02)

A majority of 17 papers from the literature list dealt with Type 02 servicescapes, such as retail stores, restaurants, and museums. Most items were categorized as product/furniture/displays (400%, *n* = 68), followed by ambient variables (282%, *n* = 48), social variables (182%, *n* = 31), and interior variables (165%, *n* = 28). It could be concluded that tangible and hedonic servicescapes are designed mainly with visible merchandise or its arrangement (composition). Moreover, adequate ambient conditions should be considered for customers to enjoy their service.

#### 4.3.4. Intangible and Utilitarian Servicescape (Type 03)

Of the 44 studies, only seven researched the Type 03 servicescape, which contains healthscape. As shown below, ambient variables (328%, *n* = 23) are the most influential dimensions, followed by social variables (214%, *n* = 15), functional variables (157%, *n* = 11), and product/furniture/displays (143%, *n* = 10). Compared with other service settings, ambient conditions and social interactions were important in healthscapes even though there was no significant difference between the functional layout and product/furniture/displays. As a utilitarian servicescape, healthcare environment clearly represented different properties from hedonic servicescape (Type 01 and Type 02). Therefore, comprehensive understanding the specific characteristics of healthscape should be considered apart from other servicescape studies. This point is discussed concretely in discussion section.

#### 4.3.5. Tangible and Utilitarian Servicescape (Type 04)

None of the studies belonged to Type 04.

#### 4.3.6. Visualization of Factor Classification 

**Figure 3:**
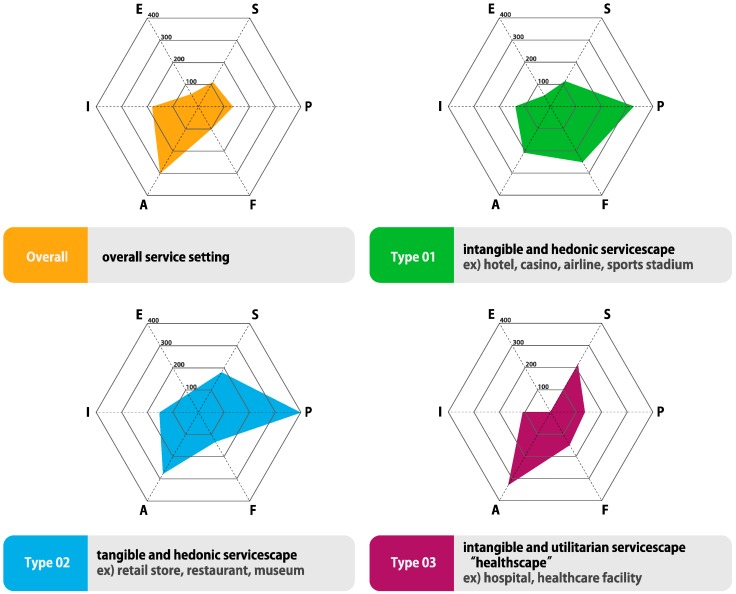
Results of factor classification by servicescape typology. *Note*: The abbreviations of the indicators are described as follows. (E): External variables, (I): Interior variables, (A): Ambient variables, (F): Functional variables, (P): Product/Furniture/Displays variables, (S): Social variables.

We summarize the results of factor classification of servicescapes and visualize them as radial graphs. Overall service, Type 01, Type 02, and Type 03 presented different features, respectively as shown in Figure 3 below. Type 03, to which healthscape belongs, is identified as requiring high levels of ambient and social factors, compared to other service settings.

### 4.4. Results and Summary

Based on a systematic literature review of the selected 44 papers, this study identified current research paths to broaden our understanding of servicescapes and healthscapes. We summarized our research findings as follows.

#### 4.4.1. Academic Terminologies and Journal Fields 

Overall, we identified 44 relevant studies in the sources that we searched, as shown in Appendix A. The term “servicescape” and “physical environment” were predominantly used to explain a tangible environment with a general service setting. Under the influence of Bitner, the neologisms “Dinescape,” “Festivalscape,” and “Healthscape” (implying the servicescape of healthcare facilities) were generated and they are currently used as official academic terminology [16,24,42].

With respect to where the servicescape literature was published, half of the studies (50%) were directly related to marketing/management/business/service fields. This contrasts strongly with the literature published in design/architecture (9%) or healthcare (6%) journals. Initially, servicescape studies were mainly implemented from the perspective of marketing or service management, although the servicescape deals with the physical environment of the service setting. The apparent lack of servicescape studies in other journal fields might be due to the fact that academic interest and attention in those fields was focused on identifying interactions between service settings and user satisfaction, and that research in this direction is less active and has low academic accessibility.

#### 4.4.2. Research Methodology 

By analyzing the research methodology of the servicescape, we found that 50% of the studies were classified as MP/DF and 11% of the studies were classified as TC. This record implied that recent studies on the servicescape tried to analyze the impact of multiple factors or integrate new frameworks based on Bitner’s study. Compared to these, healthscape studies have been approached from various angles, from theoretical analysis to framework demonstration.

#### 4.4.3. Conceptual Framework

By analyzing dependent/independent variables, we could identify the servicescape (healthscape) framework and the theoretical background that is mainly treated as a valuable concept to explain the service environment. As shown in Table 4, we found that the dominant theory on servicescape was based on service management that mainly considered user satisfaction or positive reaction to service. Most of the current studies have tried to identify relationships between servicescape cues and behavioral responses (i.e., behavioral intentions and approach/avoidance) or patronage intention (i.e., repatronage intentions, recommendations, and positive WOM). To demonstrate this, PAD (i.e., pleasure, arousal, and dominance), emotional responses (i.e., satisfaction, excitement, and affect), or service quality (i.e., perceived quality and cognition) were selected as mediating variables.

#### 4.4.4. Environmental Factors by Different Service Typology

In this study, we classified the servicescape literature by adopting the service typology by Lee et al. and compared the frequently addressed factors between different types of service settings [34]. As a result, Type 01 (i.e., intangible and hedonic) and Type 02 (i.e., tangible and hedonic) were highly related to Product/Furniture/Displays. Compared to this, Type 03 (i.e., intangible and utilitarian) and overall service were highly concerned with Ambient Variables. Type 03 that contained healthscape, especially identified that social factors are highly dealt with while evaluating healthcare facilities.

## 5. Discussion and Implications

### 5.1. Discussion and Future Challenges

This study has identified a growing body of research that examines theoretical background of the servicescape and healthscape. In relation to the outcomes, we discuss key influences and suggest future opportunities for further healthscape studies.

#### 5.1.1. The Development of a Framework and Evaluation Factors for Healthscape

Through this study, we emphasized the significance of developing criteria and framing their interactions under distinct types of service settings. Researchers have begun to understand that it is not enough to adapt the general dimensions of the servicescape to every service setting [11,38,42,43,44,45,46]. There have been few attempts to develop a theoretical background or suggest diverse frameworks that could explain how different the healthscape is from other service environments. Furthermore, there is a lack of studies to expand Bitner’s criteria to explain newly introduced service areas. Even though Hutton and Richardson created the term “healthscape” in 1995, few attempts since then have been made to investigate the healthscape. Therefore, in this study, we classified several groups of servicescapes and compared the factors that are commonly considered to evaluate the service settings. As shown in Figure 3, Type 03 (i.e., intangible and utilitarian) indicates that the healthcare facility involved represented different features compared to other service types. The results showed that researchers who studied the servicescape of Type 03 are more concerned about ambient and social factors rather than interior factors. However, these results could not be judged conclusively since the articles discussing Type 03 developed their criteria by citing other studies. In addition, even though two different service environments were involved in the same type of servicescape, there could be different servicescape factors to give customers positive feelings. For instance, adequate lighting systems would be the most significant factor in layout designs of restaurants compared to those in museums while both of them are included in Type 02. Future researchers who attempt to understand the framework of healthcare environment, therefore, should develop their own criteria according to pertinent service settings.

#### 5.1.2. A Differentiated Point of View to Evaluate Healthscape

Most of the research on the servicescape has dealt with the interaction between environmental factors and behavioral responses or patronage intentions, as shown in Table 4. These results have shown that the servicescape studies have been carried out primarily from the perspective of marketing and service management that are mainly concerned with customer satisfaction. Thus, more research is needed to identify how to evaluate physical service environments with a specific point of view. For instance, Bonfanti tried to identify customers’ needs and expectations of a servicescape surveillance management (SSM) and adapt it to three different service settings: retail, hotel, and hospital [47]. He suggested that safety, security, and privacy could be the main criteria that service managers use to design their service environments to ensure a high level of security, while enhancing the customer service experience. This implies that different servicescape factors should be developed according to the type of stakeholder (e.g., patients or employees), the type of service setting (e.g., hospital or retail), or the purpose of the service environment (e.g., customer satisfaction or surveillance). These efforts should be adapted to the research of healthscapes. For instance, healthcare environments should be especially concerned with the safety of patients. Thus, healthscapes need to be designed by considering safety aspects more important than more pleasant spaces and emotionally favorable spaces. Various environmental factors could be developed by considering the construct of healthcare. The interactions between different factors should be analyzed to understand the hidden architecture of a new service domain. According to these attempts, it is necessary to develop a more concrete framework to explain the healthcare service environments.

#### 5.1.3. Consideration on the Impact of Enhanced Technology to Healthscape 

Recent emerging technologies have expanded the area of services from physical space to cyberspace. Even further, these two distinctive spaces have merged with advanced technologies such as robotics, artificial intelligence, and cloud computing. As we previously reviewed, one of the most dramatic changes since Bitner’s study is the birth of cyberspace and the advancement in technology [20]. In this study, research on e-servicescape was intentionally excluded from the final literature list since we focused on tangible properties. The intangible characteristics of the e-servicescape should be dealt with a different perspective from physical environments because those studies purely focus on digital services in the cyberspace. Nevertheless, further studies should consider how health-related information or automated services could be delivered to customers in visible (or tangible) ways, and for this, the physical environment should be changed and improved.

Healthscape should be concerned with the co-existence of tangible and intangible environments. The advancement of Information and Communication Technologies (ICTs) has enhanced the boundaries of the physical environment on healthcare service. It is considered as a key driver in the digital transformation of healthcare industry [48]. Patients can experience healthcare service and obtain their health information without visiting a hospital or clinic. Along with these dramatic changes, further research should infuse new technological characteristics into the dimensions of healthscape to explain the newly expanded cyber-physical environment of healthcare service.

With ICTs, one of the remarkable developments that scholars should consider is the advent of nursing robots or AI doctors [31]. As electronic machines could be equipped with high levels of intelligence, robots could implement autonomous action and decision-making. The immutable object of service was human beings; however, these emerging technologies bring new stakeholders to healthcare services. Future scholars should interpret and evaluate a robot not as a smart device but as a social object that could interact with people and the environment. The former healthscape studies have discussed social factors between only the service provider and customer. However, social interactions with robots should be another noticeable issue when researchers evaluate the healthcare environment.

Such development of technology seems to offer useful and convenient healthcare service. However, a transformative potential for technology could reveal unintended negative consequences [49]. Invasion of privacy, dehumanization, and infoganda would be one of the noticeable agendas on healthcare service in the next generation. Thus, the health service experience needs to be deliberated from a holistic perspective: not only embracing traditional servicescape studies but also considering the side effects of technology. With the radical improvement of technology in healthcare services, we encourage further investigation into these challenges.

### 5.2. Implications

#### 5.2.1. Academic Implications

From the theoretical perspective, the results of this study can contribute to broaden the knowledge body of service research by conceptualizing the healthscape and by proposing a differentiated perspective from other hedonic servicescape studies. The research findings can serve as an academic compass which can support scholars to identify the academic stream and theoretical boundary of servicescape studies and to further understand the physical environment of healthcare service. The servicescape typology can allow scholars to extend research directions in diverse ways such as the comparison of significant dimensions according to different servicescape types, or the empirical verification of factor significance in each servicescape dimension.

#### 5.2.2. Managerial and Policy Implications

The results of this study also have implications for the managers and marketers of healthcare industries. The factor classifications could be referred as a guideline when managers evaluate their service environments. They can analyze strengths and weaknesses of their healthscape and have a sense of what priorities should be set to improve or renovate their facilities. Additionally, managers could use the dimension of healthscape to benchmark their major competitors. They could compare service environments with those of their business rivals by analyzing based on six dimensions of healthscape.

The research findings can propose an important direction for policy makers to regulate and to promote the healthcare industries in terms of the perspective of healthscape. Understanding different characteristics by the servicescape typology can be the basis of a distinguished assessment model for various healthcare service and service environments. Weighted value between dimensions could be applied for quality estimation in case of developing an evaluation guideline for healthcare facilities. For this purpose, a future research direction could be suggested to identify the acute weighted values of healthscape dimensions based on our research findings.

## 6. Conclusions

This study aims to fill the gap between healthscape and servicescape studies using a systematic literature review. We analyzed the academic stream on servicescape studies since Bitner and identified differing points of view between healthscape and servicescape through the classification of previous research findings. Through a rigorous and systematic literature review, we implemented an in-depth analysis of new research paths on the topic using 44 selected papers. This not only broadened the academic knowledge on servicescape but also showed practical and theoretical agendas followed in healthscape studies.

Despite these efforts, there are some limitations in our study. Due to the limited number of papers, some of the results cannot be generalized to explain the servicescape of healthcare services. In this study, we could find only seven key papers that dealt with the physical environment of healthcare services. Due to the shortage of healthscape journals, we were unable to conduct an in-depth analysis in a qualitative and quantitative way. Moreover, almost half of the healthscape studies are published in journals in the field of healthcare. More research is recommended from the point of view of the fields of psychology or design/architecture. In addition, field studies or empirical research are needed to detect which factors are significant to manage the servicescape of healthcare services, compared to the traditional servicescape framework. Furthermore, this study considered the customer interaction partially by classifying social factor as one of the primary dimensions and analyzing the framework of servicescape. Based on the results of this study, more researches should examine not only the interactions between derived servicescape factors and customer responses (e.g., physiological response, emotional response, and behavioral response) but also its delivery process.

Through this study, we suggest three points of view to consider for future healthscape studies: (1) the development of a framework and evaluation factors suitable for healthcare service settings that could be distinguished from general commercial servicescapes; (2) a differentiated point of view to evaluate the physical environment; and (3) consideration on the impact of enhanced technology to healthscape.

Our research findings can contribute to a deep understanding of new ways to interpret specific features of the servicescape and healthscape as well. Our implications are valuable as these results could suggest academic agendas in physical service environments.

## Figures and Tables

**Table 1:** Frequency analysis findings of servicescape terminology.

Term	References
Servicescape	SC04, SC06, SC11, SC16, SC17, SC18, SC21, SC24, SC29, SC30, SC31, SC32, SC35, SC37, SC38, SC39, SC40, SC42
Physical environment	SC14, SC22, SC26, SC28, SC34, SC36, SC41, SC43
Tangible (Tangserv)	SC12 (SC15)
Atmosphere	SC02, SC05, SC07, SC13, SC19, SC23
Festivalscape	SC25, SC33
Dinescape (Dineserv)	SC27 (SC10)
Healthscape	SC08, SC09, SC44

Note: underline references indicate journals on healthscape studies.

**Table 2:** Frequency analysis findings in fields of journals.

Field of Journal	References	Freq. (%)
Marketing/ Management/ Business/Service	SC02, SC04, SC05, SC06, SC08, SC09, SC11, SC12, SC13, SC14, SC15, SC16, SC19, SC21, SC23, SC24, SC25, SC26, SC27, SC29, SC31, SC32	22 (50%)
Tourism/Hotel	SC10, SC18, SC22, SC28, SC33, SC34, SC36, SC39, SC40, SC41, SC42, SC43	12 (27%)
Food/Restaurant	SC01, SC10, SC15, SC23, SC27	5 (11%)
Design/Architecture	SC30, SC35, SC37, SC38	4 (9%)
Sports/Game	SC17, SC42	2 (4%)
Psychology	SC12	1 (2%)
Retail	SC03	1 (2%)
Healthcare	SC08, SC09, SC44	3 (6%)

Notes: underline references indicate journals on healthscape studies; duplicates are allowed. Freq.: the frequency means the relevant articles compared with total reviewed literatures (*n* = 44).

**Table 3:** Frequency analysis findings regarding type of research methodology.

Research Methodology	References	Freq. (%)
TC	SC02, SC09, SC16, SC23, SC31	5 (11%)
MP	SC03, SC06, SC14	3 (6%)
DF	SC15, SC27, SC38, SC42, SC44	5 (11%)
TC/MP	SC04, SC08	2 (4%)
MP/DF	SC05, SC11, SC12, SC17, SC19, SC21, SC22, SC24, SC25, SC26, SC28, SC29, SC30, SC32, SC33, SC34, SC35, SC36, SC37, SC39, SC41, SC43	22 (50%)
TC/LR	SC13, SC18	2 (4%)

Notes: underline references are journals on healthscape studies. TC: theoretical and conceptual paper, MP: modeling papers, DF: dimension/factor analysis, LR: literature review. Freq.: the frequency means the relevant articles compared with total reviewed literatures (*n* = 44).

**Table 4:** Framework of servicescapes (frequencies of independent, mediating and moderating variables).

Journal Index	PAD	ER	SQ	LI	I	RE	BR	PI
SC03							+	
SC04						+	+	
SC05			(+)		+			
SC06		(+)						+
SC08						+	+	
SC11		(+)	(+)			+	+	
SC12		(+)	(+)					+
SC14			(+)				+	
SC17		(+)				+		+
SC19							+	+
SC21	(+)						+	
SC22	(+)						+	
SC24				+				
SC25		(+)		+				
SC26	(+)						+	
SC28		(+)		+		(+)		
SC29	(+)							+
SC30		+	+					+
SC31							+	
SC32		(+)					+	
SC33		(+)					+	
SC34		(+)				+		+
SC35		(+)	(+)	+				+
SC36		+			(+)			+
SC37							+	
SC39	+		+		+			
SC40								+
SC41	+	+						
SC43	(+)	+						

Notes: Examples of aggregation of the variables follow. PAD: pleasure, arousal, dominance, etc. ER (emotional responses): satisfaction, excitement, affect, etc. SQ (service quality): perceived quality and cognition. LI (loyalty intentions): loyalty I (image): store evaluation, store environment, etc. RE (resource expenditure): money/time spent, desire to stay, etc. BR (behavioral responses): behavioral intentions, approach/avoidance behavior, etc. PI (patronage intention): repatronage intentions, recommendation, positive WOM, etc. (+): mediating/moderating variables; +: independent variables.

**Table 5:** Factor classification by the servicescape typology.

**Overall Service Setting**								
**ID**	**Factors**	**Attributes/Items**	**(E)**	**(I)**	**(A)**	**(F)**	**(P)**	**(S)**
SC01	1. Architecture 2. Lighting 3. Temperature 4. Furnishings 5. Layout 6. Color		+	+	++	+	+	
SC02	1. Ambient factor 2. Design factor 3. Social factor	1. air quality, noise, scent, cleanliness 2. aesthetic (architecture, color, scale, material, texture, shape, style, accessory), functional (layout, comfort, signage) 3. other customer (number, appearance, behavior), service personnel (number, appearance, behavior)	+	+++ ++	+++ ++	++	++	+++ +++
SC04	1. Ambient factors 2. Spatial layout and functionality 3. Signs, symbols and artifacts	1. temperature, air quality, noise, music, and odor 2. layout, equipment, and furnishings 3. signage, personal artifacts, and style of décor		+	+++ ++	+	+++ +	
SC18	1. Visual cues 2. Auditory cues 3. Olfactory cues	1. color, space and function, lighting 2. music, non-musical sound		+	+++	+		
SC20	1. Facility exterior 2. Facility interior		+	+				
Total (*n* = 5)			3 (60%)	9 (180%)	15 (300%)	5 (100%)	7 (140%)	6 (120%)
**(Type 01) Intangible and Hedonic Servicescape**								
**ID**	**Factor**	**Attributes/Items**	**(E)**	**(I)**	**(A)**	**(F)**	**(P)**	**(S)**
SC06	1. Spatial layout and functionality 2. Aesthetics	1. stadium seats, ticket windows/gates, hallways/walkways, entrances/exits, food service areas, and rest-rooms 2. external environment, exterior construction, interior construction, score-boards, and facility cleanliness	++	+	+	+++ +++ ++	++	
SC11	1. Layout accessibility 2. Facility aesthetics 3. Seating comfort 4. Electronic equipment/displays 5. Facility cleanliness	1. layout of exit and entry, furnishing, and equipment layout 2. architectural design, color, and interior design 3. physical seat and space of seat 4. signs, symbols, and artifacts for leisure experience 5. facility cleanliness	+	++	+	+	+++ +++ +	
SC12	1. Building design and décor 2. Equipment 3. Ambience	1. outside appearance, interior design, layout, seats 2. electric equipment 3. cleanliness, temperature, and neatness of employees‘ appearance	+	+	++	+	++	+
SC14	1. Servicescape	1. enough employee, enough space, superior interior environment, smell, lighting, cleanliness, comfortable physical facility, pleasing interior layout, color, material, interior accessory		+++ +		+	++	+
SC17	1. Ambient factor 2. Layout/Navigation 3. Cleanliness 4. Interior décor 5. Seating comfort	1. architecture, interior design, and spatial layout 2. clean slot door 3. padding, back rests, fabric/heat-dissipating seat, and uncrowded seat 4. lighting, color, and door décor 5. internal climate, music, and visual graphic	+	+++	+++ +	+	+++ ++	+
SC21	1. Space 2. Way-findings	1. spatial density 2. legibility of internal design such as central passageways and meeting places				+	+	+
SC25	1. Program contents 2. Staff 3. Facility 4. Food 5. Souvenir 6. Convenience 7. Information	1. skip (regardless of this study) 2. kind guides and staff, quick responsiveness on request, willingness to help, enough knowledge, courteous guides and staff, number of event guides and staff 3. comfortable festival sites, facility, space/size, cleanliness, atmosphere of site, layout 4. quality, price, available traditional food, variety 5. variety, quality, price 6. restroom, parking lot, rest area 7. installed signboards, prepared pamphlets, total variance explained		++	+	+++ ++	+++ +++	+++ +++
SC32	1. Ambient factor 2. Functional factor 3. Aesthetic factor 4. Safety factor 5. Social factor	1. humidity, circulation, temperature, light, scent, noise 2. comfort, flow of traffic, signage, size, layout, arrangement 3. color, accessory, decoration, architecture, materials 4. fire equipment, safety sign, hazard detector, antiskid tool 5. other customer (number, appearance, behavior), service personnel (number, appearance, behavior)	+	+++	+++ ++	+++ +	+++ +++ +	+++ +++
SC33	1. Fun 2. Food 3. Comfort	1. food quality, beverage quality 2. promotional activity, live entertainment, time of scheduled events, printed information, exhibition and trade stands, signposting, helpfulness of staff 3. feeling of safety, cleanliness of restroom, number of seats, cleanliness of sites, accessibility for elderly/disabled and children, accessibility to public toilets		++		++	+	
SC34	1. Ambient onditions 2. Space/Function	1 Air quality, temperature, odor, noise 2. layout, equipment/amenity			+++ +	+	++	
SC35	1. Attractiveness 2. Cleanliness 3. Layout 4. Comfort	1. Finishes, colors, and facilities 2. Clean facilities, entrances, corridors, and restrooms 3. Easy access to designated places 4. Comfortable air, temperature, and brightness		+++	+++	+++ +	+	
SC36	1. Design 2. Equipment 3. Environment 4. Ambience 5. Social Factors			+	+	+	+	+
SC37	1. Cleanliness 2. Layout 3. Facility aesthetics 4. Ambience	1. cleanliness 2. accessibility, layout 3. exterior design style, interior design style, color, finishing material, decoration, furniture and lighting 4. temperature, lighting, air quality	+	+++ +	+++ +	++	+	
SC41	1. Layout accessibility 2. Facility ambience and aesthetics 3. Functionality 4. Cleanliness	1. sign, baggage trolley, layout for people (elderly, pregnant woman), location, accessibility 2. color, decoration, brightness, temperature, music, aroma 3. spacious seating, signs, electronic facility, internet connectivity, charging device, electronic walkway		+	+++ +	+++	+++ +++ +++	
SC43	1. Layout Accessibility 2. Facility Aesthetics 3. Functionality 4. Cleanliness	1. layout 2. color, attractive character, fashion, attractive facility 3. waiting area, seating, electronics displays information, high-quality displays, exciting electronic facility 4. restroom, food service area, clean		+++ +	+	+++ +	+++	
Total (*n* = 15)			7 (47%)	21 (140%)	31 (207%)	39 (260%)	50 (333%)	17 (113%)
**(Type 02) Tangible and Hedonic Servicescape**								
**ID**	**Factor**	**Attributes/Items**	**(E)**	**(I)**	**(A)**	**(F)**	**(P)**	**(S)**
SC03	1. Ambient factor 2. Social factor				+			+
SC05	1. Ambient factors 2. Design factors 3. Social factors	1. music, lighting 2. color, brass trim on displays, layout, organization of merchandise 3. number of salesperson, greeting by salesperson, salesperson dress		+	++	+	++	+++
SC07	1. Store exterior 2. General interior 3. Layout and design 4. Point-of-purchase 5. Decoration ariables		+	+		+	+	
SC10	1. Reliability 2. Responsiveness 3. Empathy 4. Assurance 5. Tangibles						+	
SC13	1. External variables 2. General interior variables 3. Layout and design variables 4. Point of purchase and decoration variables 5. Human variables	1. store front, marquee, entrance, display windows, building architecture, parking, surrounding area 2. flooring/carpeting, lighting, scent, sounds, temperature, cleanliness, fixtures, wall coverings, cash register placement 3. floor Space allocation, product groupings, traffic flow, department locations, allocations within department 4. product displays, racks and cases, posters, signs, cards, wall decorations 5. crowding, customer characteristics, employee characteristics, employee uniforms	+++ ++++	+++ +	+++ ++	+++ ++	+++ +++ +	+++ +
SC15	1. Layout/design factors 2. Product/service factors 3. Ambient/social factors	1. interior decoration, building design, dining hall size, restaurant location, seating arrangement 2. food presentation, size of food serving, Menu design, food variety 3. light, crowding, music, temperature	+	+	+++	++	+++ ++	+
SC19	1. Exterior factors 2. Interior factors 3. Layout and design 4. Decorations 5. Human factors		+	+		+	+	+
SC22	1. Facility aesthetics 2. Lighting 3. Ambience 4. Layout 5. Dining equipment 6. Employees	1. attractive paintings and/or pictures, appealing wall décor, beautiful plants and/or flowers, warm colors, high quality furniture 2. warm lighting, welcoming lighting, comfortable lighting 3. relaxing music, pleasing music, comfortable temperature, enticing aroma 4. enough seat space, crowded seating arrangement, easy-to-move layout 5. high quality tableware, attractive linens, attractive table setting 6. attractive employees, neat and well-dressed employees		++	+++ +		+++ +++ +++	++
SC23	1. Internal variables 2. Layout and design 3. Human variables 4. Social facilitation	1. music, noise, and odor 2. table layout and seating 3. density and crowding 4. eating alone or with others			+++		++	+++
SC24	1. Ambient condition 2. Design factors 3. Staff behavior 4. Staff image	1. music, aroma, cleanliness 2. implicit communications, furnishing 3. customer orientation, credibility 4. competence, physical attractiveness			+++		+	+++ +++
SC26	1. Facility aesthetics 2. Lighting 3. Ambience 4. Layout 5. Dining equipment 6. Employees	1. attractive paintings and/or pictures, appealing wall décor, beautiful plants and/or flowers, warm colors, high quality furniture 2. warm lighting, welcoming lighting, comfortable lighting 3. relaxing music, pleasing music, comfortable temperature, enticing aroma 4. enough seat space, crowded seating arrangement, easy-to-move layout 5. high quality tableware, attractive linens, attractive table setting 6. attractive employees, neat and well-dressed employees		++	+++ +		+++ +++ +++	++
SC27	1. Facility Aesthetics 2. Ambience 3. Lighting 4. Table settings 5. Layout 6. Service staff	1. attractive paintings and/or pictures, appealing wall décor, beautiful plants and/or flowers, warm colors, high quality furniture 2. relaxing music, pleasing music, comfortable temperature, enticing aroma 3. warm lighting, welcoming lighting, comfortable lighting 4. high quality tableware, attractive linens, attractive table setting 5. enough seat space, crowded seating arrangement, easy-to-move layout 6. attractive employees, neat and well-dressed employees		++	+++ +		+++ +++ +++	++
SC28	1. Decor and artifacts 2. Spatial layout 3. Ambient conditions	1. painting/pictures, plant/flower, ceiling décor, wall décor, color, furniture 2. layout, table/seating arrangement 3. lighting, music, temperature, aroma		+++	+++ +	+	+++ +++	
SC29	1. Ambience ondition 2. Facility aesthetics 3. Layout 4. Electric equipment 5. Seating comfort	1. lighting level, temperature, aroma, and background music 2. architecture, interior, décor, color, and overall attractiveness 3. tables, service areas, and passageways 4. audio/video equipment 5. comfortable seat and uncrowded seat	+	+++ +	+++ +	++	+++ ++	
SC38	1. Aesthetics 2. Ambient 3. Space/Function 4. Seating comfort 5. Cleanliness	1. architecture, interior décor, style, color 2. temperature, aroma, music, lighting 3. seating space, aisle space, sign, tableware 4. comfortable distance, seat accessibility, comfortable seats 5. employee, restroom, overall cleanliness	+	+++ ++	+++ +	++	+++ ++	+
SC40	1. Physical setting 2. Service provider 3. Other customers					+		++
SC42	1. Accessibility/Convenience 2. Signage 3. Facility layout 4. Design 5. Equipment/facility condition 6. Ambient 7. Facility system 8. Social	1. parking, accessibility, location 2. sign 3. large, design for all level of ability, enough space, waiting time 4. layout, fashion, attractive, layout 5. high quality, working condition, well equipped, safe, maintenance 6. noise, cleanliness, comfortable, music 7. light, temperature, air quality, heating/ventilation 8. employee (helpful, friendly), customer (helpful, friendly)	++	++	+++ +++ +	+++ +++ +	+++ ++	+++ +
Total (*n* = 17)			14 (82%)	28 (165%)	48 (282%)	23 (135%)	68 (400%)	31 (182%)
**(Type 03) Intangible and Utilitarian Servicescape**								
**ID**	**Factor**	**Attributes/Items**	**(E)**	**(I)**	**(A)**	**(F)**	**(P)**	**(S)**
SC08	1. Ambient conditions 2. Space/function 3. Signs, symbols, artifacts				+	+	+	
SC09	-							
SC16	1. Ambient factor 2. Design factor 3. Social interaction factor	1. ambient items 2. aesthetic, functional 3. customer, Employee		+	++	+		++
SC30	1. Ambient factor 2. Serviceability factor	1. temperature, air quality, acoustics, visual attractiveness, lighting, furniture 2. convenience in layout, privacy, communication staff, way-finding, cleanliness		+	+++ ++	++	+	++
SC31	1. Restorative dimension 2. Physical dimension 3. Social dimension	1. being away, fascination, compatibility 2. ambient condition: temperature, air quality, noise, music, odor space/function: layout, equipment, furnishings signs/symbols/artifacts: signage, artifacts, style of decor 3. employees, members, emotional contagion		+	+++ ++	+	+++ +	+++
SC39	1. Ambient factor 2. Design factor 3. Social factor	1. odor, light, cleanliness, temperature, music, noise 2. rest room, color, quality, attractive interior, picture 3. employee (dressed, helpful, friendly, number), customer appearance		++	+++ +++	+	+	+++ ++
SC44	1. Service personnel conduct and cleanliness 2. Service delivery 3. Ambience and facilities 4. Location and look 5. Appealing decoration 6. Upgraded safety service	1. staff care, employee behavior, staff welcome, laundry service, corridor cleanliness, ambulance service, toilet cleanliness 2. care room, recovery room, waiting chair, waiting area 3. appealing physical facility, color, furnishing, attractive layout 4. lighting, location, spacious look 5. peer group visit, appealing entrance decor, odor, noise 6. safety and security measures, medical equipment update		+++	+++ +	+++ ++	+++	+++
Total (*n* = 7)			0 (0%)	8 (114%)	23 (328%)	11 (157%)	10 (143%)	15 (214%)

Notes: Examples of aggregation that dependent variables follow. (E): External variables, (I): Interior variables, (A): Ambient variables, (F): Functional variables, (P): Product/Furniture/Displays, (S): Social variables. +: an item which could be relevant to each servicescape’s dimension. Total amount of “+” is counted in “Total” row by each dimension.

## Appendix A

**Table A1:** Journals included in this review.

ID	Author	Year	Terminology Used	Journal Name	Journal Type	Service Typology	Data Collection Methods/Sample
SC01	Booms et al. [50]	1982		*Cornell Hotel and Restaurant Administration Quarterly*			
SC02	Baker [29]	1986	Atmosphere	*The services Challenge*	TC	Overall service setting	
SC03	Baker et al. [51]	1992	Environment, Physical Features	*Journal of Retailing*	MP	Type 02	Self-reporting survey and video recording analysis/American retail store (*n* = 147)
SC04	Bitner [2]	1992	Servicescape	*The Journal of Marketing*	TC, MP	Overall service setting	
SC05	Baker et al. [52]	1994	Store Atmosphere	*Journal of the Academy of Marketing Science*	MP, DF	Type 02	Self-reporting survey/USA Retail customers (undergraduate students) (*n* = 297)
SC06	Wakefield et al. [3]	1994	Servicescape	*Journal of Services Marketing*	MP	Type 01	Self-reporting survey/five leisure stadium customers (*n* = 3900)
SC07	Berman et al. [53]	1995	Atmosphere			Type 02	
SC08	Hutton et al. [16]	1995	Healthscapes	*Healthcare Management Review*	TC, MP	Type 03	Literature review/healthcare service setting
SC09	Hutton et al. [17]	1995	Healthscapes	*Marketing Health Services*	TC	Type 03	Literature review/healthcare service setting
SC10	Steven et al. [54]	1995	DINESERV	*Cornell Hotel and Restaurant Administration Quarterly,*		Type 02	Restaurant
SC11	Wakefield et al. [30]	1996	Servicescape	*Journal of Services Marketing*	MP, DF	Type 01	Self-reporting survey/American leisure service setting (football, baseball, and casino) (*n* = 1263)
SC12	Wakefield et al. [55]	1999	Tangible Service Factors	*Psychology & Marketing*	MP, DF	Type 01	Self-reporting survey/USA leisure service setting (professional hockey games, a family recreation center, movie theater) (*n* = 260,115,124)
SC13	Turley et al. [21]	2000	Atmosphere	*Journal of Business Research*	TC, LR	Type 02	
SC14	Hightower et al. [4]	2002	Physical environment	*Journal of Business Research*	MP	Type 01	Self-reporting survey/American baseball stadium (*n* = 125)
SC15	Raajpoot [12]	2002	TANGSERV	*Journal of Foodservice Business Research*	DF	Type 02	Self-reporting survey/restaurants Users (*n* = 234)
SC16	Hightower [56]	2003	Servicescape	*Marketing Management Journal*	TC	Type 03	Literature review/American funeral service provider *
SC17	Lucas [5]	2003	Servicescape	*UNLV Gaming Research & Review Journal*	MP, DF	Type 01	Self-reporting survey/American hotel casino (*n* = 195)
SC18	Lin [6]	2004	Servicescape	*International Journal of Hospitality Management*	TC, LR	Overall service setting	
SC19	Kottasz [7]	2006	Atmospheric Cue	*Journal of Nonprofit and Public Sector Marketing*	MP, DF	Type 02	Self-reporting survey/UK Museum visitors (*n* = 140)
SC20	Zeithaml et al. [8]	2006					
SC21	Newman [15]	2007	Servicescape	*The Service Industries Journal*	MP, DF	Type 01	Self-reporting survey/international airport (*n* = 100)
SC22	Ryu et al. [13]	2007	Physical environment	*Journal of Hospitality & Tourism Research*	MP, DF	Type 02	Self-reporting survey/USA Upscale restaurants (*n* = 253)
SC23	Edwards et al. [57]	2008	Atmosphere	*Journal of Foodservice*	TC	Type 02	Restaurants
SC24	Harris et al. [14]	2008	Servicescape	*European Journal of Marketing*	MP, DF	Type 02	Self-reporting survey/UK restaurant patrons (*n* = 271)
SC25	Lee et al. [27]	2008	Festivalscapes	*Journal of Business Research*	MP, DF	Type 01	Self-reporting survey/Korean Dance festival participants (*n* = 472)
SC26	Ryu et al. [9]	2008a	Physical Environment	*The Service Industries Journal*	MP, DF	Type 02	Self-reporting survey/USA Upscale restaurants (*n* = 253)
SC27	Ryu et al. [42]	2008b	Dinescape	*Journal of Foodservice Business Research*	DF	Type 02	Self-reporting survey/USA three Upscale restaurants (*n* = 319)
SC28	Han et al. [39]	2009	Physical Environment	*Journal of Hospitality & Tourism Research*	MP, DF	Type 02	Self-reporting survey/USA restaurants customers (*n* = 279)
SC29	Kim et al. [38]	2009	Servicescape	*International journal of hospitality management*	MP, DF	Type 02	Self-reporting survey/Canada theme restaurants (*n* = 208)
SC30	Lee [18]	2011	Servicescape	*International Journal of Design*	MP, DF	Type 03	Self-reporting survey/American student healthcare clinic (*n* = 151)
SC31	Rosenbaum et al. [19]	2011	Servicescape	*Managing Service Quality*	TC	Type 03	conceptual paper/American cancer resource center
SC32	Jeon et al. [58]	2012	Servicescape	*Service Business*	MP, DF	Type 01	Self-reporting survey/South Korea international airport passengers (*n* = 290)
SC33	Mason et al. [59]	2012	Festivalscape	*Tourism Management*	MP, DF	Type 01	Self-reporting survey/Italian food and wine festival visitors (*n* = 380)
SC34	Han [10]	2013	Physical environment	*Tourism Management*	MP, DF	Type 01	Self-reporting survey/Multiple Asian Countrieslow cost airlines passengers (*n* = 331)
SC35	Lee et al. [43]	2014	Servicescape	*Journal of Asian Architecture and Building Engineering*	MP, DF	Type 01	Self-reporting survey/Korean five public service facilities (community center, sports complex, youth center, cultural center) (*n* = 594)
SC36	Wu et al. [60]	2014	Physical environment	*Journal of Hospitality and Tourism Research*	MP, DF	Type 01	Self-reporting survey/Taiwan theme parks customers (*n* = 424)
SC37	Lee et al. [45]	2015	Servicescape	*Journal of Asian Architecture and Building Engineering*	MP, DF	Type 01	Self-reporting survey/South Korea Hotel consumers (*n* = 202)
SC38	Lee et al. [44]	2015	Servicescape	*Journal of Asian Architecture and Building Engineering*	DF	Type 02	Self-reporting survey/Taiwan theme restaurant consumers (*n* = 279)
SC39	Loureiro [61]	2015	Servicescape	*Current Issues in Tourism*	MP, DF	Type 03	Self-reporting survey/Portugal private hospital patients (*n* = 332)
SC40	Wang et al. [40]	2015	Servicescape	*Journal of Hospitality and Tourism Research*	-	Type 02	Online Self-reporting survey/USA and ethnic Chinese restaurants Users (*n* = 274,275)
SC41	Ali et al. [11]	2016	Physical environment	*Tourism Management*	MP, DF	Type 01	Self-reporting survey/Malaysia International airport Users (*n* = 271)
SC42	Kim et al. [46]	2016	Servicescape	*International Journal of Sport Management Recreation & Tourism*	DF	Type 02	Self-reporting survey/USA Fitness center Users (*n* = 178)
SC43	Moon et al. [62]	2016	Physical Environment	*Asia Pacific Journal of Tourism Research*	MP, DF	Type 01	Self-reporting survey/Korean airports passengers (*n* = 214)
SC44	Sahoo et al. [20]	2016	Healthscape	*International Journal of Health Care Quality Assurance*	DF	Type 03	Self-reporting survey/India private hospital patients (*n* = 378)

Note: lists a Table e numbered in chronological order. *: journal studied at the service provider’s viewpoint.
